# Enhanced sphingosine-1-phosphate levels ameliorate murine septic cardiomyopathy

**DOI:** 10.1186/2197-425X-3-S1-A615

**Published:** 2015-10-01

**Authors:** SM Coldewey, E Benetti, M Collino, M Bauer, A Huwiler, C Thiemermann

**Affiliations:** University Hospital Jena, Anaesthesiology & Intensive Care Medicine, Jena, Germany; University Hospital Jena, Center for Sepsis Care and Control, Jena, Germany; Department of Drug Science & Technology, University of Turin, Turin, Italy; Institute of Pharmacology, University of Bern, Bern, Switzerland; Queen Mary University of London, William Harvey Research Institute, London, United Kingdom

## Introduction

Sphingosine-1-phosphate (S1P) regulates distinct biological functions by binding to S1P receptors S1PR_1-5_ and is generated by phosphorylation of sphingosine via sphingosine kinases (SPHK) 1 and 2. The S1P mimetic FTY720 acts in its phosphorylated isoform as an unselective agonist on S1PR_1_ and S1PR_3-5_ and a selective functional antagonist on S1PR_1_ ([1]). The role of the S1P-S1PR signalling axis in the pathophysiology of septic cardiomyopathy is not known.

## Objectives

Here we investigate whether pharmacological or genetic approaches to alter S1P serum levels may attenuate sepsis-induced cardiac dysfunction in mice.

## Methods

Sepsis-associated cardiac dysfunction was mimicked by co-administration of the bacterial cell-wall components lipopolysaccharide (LPS; 9 mg/kg *i.p.*) and peptidoglycan (PepG; 1 mg/kg bw *i.p.*) in wild-type (WT) and sphingosine kinase 2 deficient mice (SPHK2^-/-^) ([2]). At 1 h after LPS/PepG mice received FTY720 (0.1 mg/kg bw *i.v.)*. To elucidate the mechanisms underlying the observed effects of FTY720 mice were treated with a selective phosphatidylinositol 3 kinase (PI3K) inhibitor (LY294002; 0.3 mg/kg bw *i.v,*) or a selective S1P_2_ receptor antagonist (JTE013; 1 mg/kg bw *i.v.*) prior to FTY720. 18 h after LPS/PepG challenge cardiac function was assessed by echocardiography, serum S1P was measured by liquid chromatography-coupled tandem mass spectrometry and expression of selected signalling molecules was determined by immunoblot analysis.

## Results

Compared to sham, mice subjected to LPS/PepG demonstrated a significant reduction in EF, indicating impaired left ventricular systolic contractility, as well as a significant decrease of serum S1P. In SPHK2^-/-^ mice, which have higher endogenous S1P levels, LPS/PepG-induced reduction of EF was significantly lower than in WT mice. Treatment with FTY720 significantly attenuated the impaired EF in WT mice and was accompanied by a significant increase of serum S1P as well as an increased phosphorylation (activation) of AKT und endothelial nitric oxide synthase (eNOS) in heart tissue. The protective effects of FTY720 were abolished following co-administration of either a PI3K inhibitor or a S1PR_2_ antagonist. Similar cardioprotective effects of FTY720 were seen in mice with sepsis caused by cecal ligation and puncture.

## Conclusions

We show here for the first time that the impaired left ventricular systolic contractility caused by LPS/PepG in WT mice is attenuated by FTY720 (given 1 h after LPS/PepG) and that this effect is accompanied by increased serum S1P levels. Mechanistically, our results indicate that activation of S1PR_2_ by increased serum S1P and the subsequent activation of the PI3K signalling axis contribute to the observed cardioprotective effect of FTY720 in experimental sepsis.
